# Synthesis and Biological Evaluation of Hydrazone Derivatives as Antifungal Agents

**DOI:** 10.3390/molecules20059229

**Published:** 2015-05-20

**Authors:** Bruna B. Casanova, Mauro N. Muniz, Thayse de Oliveira, Luís Flavio de Oliveira, Michel M. Machado, Alexandre M. Fuentefria, Grace Gosmann, Simone C. B. Gnoatto

**Affiliations:** 1 Laboratório de Fitoquímica e Síntese Orgânica (LAFIS), Faculdade de Farmácia, Universidade Federal do Rio Grande do Sul (UFRGS), Porto Alegre 90610-000, RS, Brazil; E-Mails: mauro.neves.muniz@gmail.com (M.N.M.); grace.gosmann@ufrgs.br (G.G.); 2 Laboratório de Micologia Aplicada, Departamento de Analises, Faculdade de Farmácia, Universidade Federal do Rio Grande do Sul (UFRGS), Porto Alegre 90610-000, RS, Brazil; E-Mails: thayseoliveira-@hotmail.com (T.O.); alexandre.fuentefria@ufrgs.br (A.M.F.); 3 Departamento de Farmácia, Universidade Federal do Pampa, Km 592, Uruguaiana 97500-970, RS, Brazil; E-Mails: tcheluisoliveira-@gmail.com (L.F.O.); michelmachado@unipampa.edu.br (M.M.M.)

**Keywords:** aldehydes, hydrazones, emerging yeasts, antifungal activity

## Abstract

Emerging yeasts are among the most prevalent causes of systemic infections with high mortality rates and there is an urgent need to develop specific, effective and non-toxic antifungal agents to respond to this issue. In this study 35 aldehydes, hydrazones and hydrazines were obtained and their antifungal activity was evaluated against *Candida* species (*C. parapsilosis*, *C. tropicalis*, *C. krusei*, *C. albicans*, *C. glabrata* and *C. lusitaneae*) and *Trichosporon asahii*, in an *in vitro* screening. The minimum inhibitory concentrations (MICs) of the active compounds in the screening was determined against 10 clinical isolates of *C. parapsilosis* and 10 of *T. asahii*. The compounds 4-pyridin-2-ylbenzaldehyde] (**13a**) and *tert*-butyl-(2*Z*)-2-(3,4,5-trihydroxybenzylidine)hydrazine carboxylate (**7b**) showed the most promising MIC values in the range of 16–32 μg/mL and 8–16 μg/mL, respectively. The compounds’ action on the stability of the cell membrane and cell wall was evaluated, which suggested the action of the compounds on the fungal cell membrane. Cell viability of leukocytes and an alkaline comet assay were performed to evaluate the cytotoxicity. Compound **13a** was not cytotoxic at the active concentrations. These results support the discovery of promising candidates for the development of new antifungal agents.

## 1. Introduction

Since the 1980s yeasts have emerged as some of the main agents of nosocomial systemic infections, particularly in immunocompromised people or those who are undergoing intensive chemotherapy [1,2]. Approximately two decades ago *Candida albicans* represented 70%–80% of the clinical isolates from fungaemia cases [3,4], but in the last years this has been changing. In 2011 for example the prevalence of candidemia by non-*albicans* species of *Candida* in the world was about 50% [5]. Moreover, the most frequent species of non-*albicans* *Candida* are *C. glabrata*, *C. parapsilosis* and *C. tropicalis* being the *C. parapsilosis* the third most common species in clinical isolates [5,6,7]. *Trichosporon* is considered the second most common genus cause of fungaemia in patients with hematologic malignant diseases, being besides resistant to amphotericin and echinocandins.

Thereby, the emergent pathogenic species associated with the increase of antifungal resistance have become a global problem for the success of the therapy against these microorganisms. In addition, most of the antifungals used in fungaemia cases have several adverse effects and high costs [8].

In this context, compounds containing hydrazine and hydrazone functional groups are widely studied due to their described tuberculostatic [9], anticonvulsant [10], analgesic, anti-inflammatory [11], antiplatelet [12], antifungal [13], antiviral [14], antitumor [15] and antimalarial activities [16]. Thus, according to Narang *et al.* [16], hydrazones constitute an important class of compounds for the development of new chemical entities to treat various diseases of clinical importance and besides presenting these interesting biological applications, they are easily synthesized in good yields.

Considering the clinical relevance of fungal infections and the broad biological activity demonstrated for hydrazines and hydrazones, this study aimed to synthesize a series of compounds containing these chemical functions and evaluate their antifungal activity against emerging species of *C. parapsilosis* and *T. asahii*, their mechanism of action and *in vitro* cytotoxicity.

## 2. Results and Discussion

### 2.1. Synthesis

Twenty-eight compounds were synthesized: seven aldehydes, 14 hydrazones and seven hydrazines, through a synthetic route that employs two steps from the aldehydes. All synthesized compounds are shown in Table 1.

**Table 1:** All compounds tested in this work ^a^.

		R		R		R
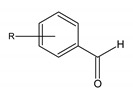	**1a** ^b^	H	**6a** ^b^	3,5-OH	**11a**	3,5-OMe
	**2a** ^b^	3-OH	**7a** ^b^	2,3,4-OH	**12a**	2,3,4-OMe
	**3a** ^b^	4-OH	**8a**	3-OMe	**13a**	4-(2)pyridinyl)
	**4a** ^b^	4-Cl	**9a**	4-OMe	**14a**	4-(4)NO_2_benzyl
	**5a** ^b^	2,4-OH	**10a**	2,4-OMe	****	
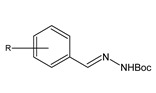	**1b**	H	**6b** ^c^	3,5-OH	**11b**	3,5-OMe
	**2b**	3-OH	**7b**	2,3,4-OH	**12b**	2,3,4-OMe
	**3b**	4-OH	**8b**	3-OMe	**13b**	4-(2)pyridinyl)
	**4b**	4-Cl	**9b**	4-OMe	**14b** ^c^	4-(4)NO_2_benzyl
	**5b**	2,4-OH	**10b**	2,4-OMe		
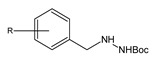	**1c**	H	**10c**	2,4-OMe	**13c**	4-(2)pyridinyl)
	**8c**	3-Ome	**11c**	3,5-OMe		11
	**9c**	4-Ome	**12c**	2,3,4-OMe		12

^a^ A total of 35 compounds were screened, seven commercially available ones purchased from Sigma-Aldrich Co. (St. Louis, MO, USA) ^b^ and 28 prepared by us. ^c^ Unpublished compounds; the corresponding structural elucidation data are presented in the Experimental Section. The other products are known compounds and were identified by comparison with authentic samples.

The aldehydes **8a–12a** were synthesized by *O*-methylation of the hydroxylated aldehydes **2a–3a**, **5a–7a**. Compounds **13a** and **14a** were obtained by Suzuki coupling between the appropriate boronic acid and the brominated aryl compound. The aldehydes **1a–14a** were subjected to coupling with *tert*-butyl carbazate, forming the corresponding hydrazones. The final step was the reduction of these hydrazones, which led to the hydrazines **1c** and **8c–13c**. The hydrazines produced from the hydrazones **2b–7b** and **14b** could not be analyzed due to the low yields.

### 2.2. Antifungal Activity

All the compounds shown in Table 1 were subjected to an antifungal activity screening at the concentration of 500 μg/mL against seven clinical isolates: *C. albicans*, *C. krusei*, *C. parapsilosis*, *C. tropicalis*, *C. glabrata*, *C. lusitaneae* and *T. asahii*. The active compounds of each class tested in the screening are shown in Table 2.

**Table 2:** Active compounds of each class at the concentration of 500 μg/mL.

Column Heading	Aldehydes	Hydrazones	Hydrazines
*C. glabrata*	**2a, 5a, 7a, 13a, 14a**	**7b, 12b**	**12c**
*C. parapsilosis*	**2a, 6a, 7a, 8a, 10a, 11a, 13a**	**2b, 5b, 6b, 7b, 11b, 13b**	**10c, 13c**
*C. tropicalis*	**2a, 5a, 8a, 10a, 12a, 13a, 14a**	**7b, 8b, 11b, 14b**	**13c**
*C. krusei*	**2a, 5a, 12a, 13a, 14a**	**7b, 9b, 12b**	**8c**
*C. lusitaneae*	**2a, 4a, 6a, 13a, 14a**	**2b, 6b, 7b, 9b, 11b**	**13c**
*C. albicans*	**2a, 5a, 7a, 10a, 11a, 13a**	**7b, 10b, 11b, 13b**	**1c, 9c, 10c, 13c**
*T. asahii*	**1a, 2a, 5a, 6a, 8a, 13a**	**3b, 5b, 6b, 7b, 11b, 13b**	**9c, 10c, 12c, 13c**

Therefore, 30 compounds showed inhibitory activity against at least one species. The compounds **2a**, **13a** and **7b** inhibited the growth of all species tested, while **3a**, **9a**, **11b**, **4b** and **11c** did not inhibit the growth of any. The species that showed the greatest susceptibility were *T. asahii*, *C. parapsilosis* and *C. albicans*. *C. parapsilosis* is described as the third most frequently found species of non-*albicans* *Candida* in clinical isolates [5,6,7] and *Trichoporon* as the second most important genus (only behind *Candida*) [5]. Therefore, the compounds that showed activity against *C. parapsilosis* (compounds **2a**, **6a–8a**, **10a**, **11a**, **13a**, **2b**, **5b–7b**, **11b**, **13b**, **10c** and **13b**) and *T. asahii* (compounds **1a**, **2a**, **5a**, **6a**, **8a**, **13a**, **3b**, **5b–7b**, **11b**, **13b**, **9c**, **10c**, **12c** and **13c**) in the screening were selected for MIC determination. The MIC assay was performed against 10 clinical isolates of each species (*T. asahii* and *C. parapsilosis*). The aldehyde **13a** and the hydrazone **7b** stood out for presenting MIC values <125 μg/mL against both species tested. These results are shown in Table 3. The others compounds tested showed MIC values between 125 μg/mL and 500 μg/mL. The variability of the responses can be due to the different susceptibility profiles the isolates showed in previous studies [17]. Therefore, in such cases, mechanisms of resistance may be constantly expressed, which results in these different profiles.

**Table 3:** Minimum inhibitory concentration (MIC) in µg/mL of the compounds **13a** and **7b**, selected from the initial screening, against clinical isolates.

Isolate	*T. asahii*			Isolate	*C. parapsilosis*		
	13a	7b	Fluc ^a^		13a	7b	Fluc
TAH 05	125	16	Nt ^b^	RL 01	32	16	64
TAH 06	64	16	32	RL 05	32	16	≤1
TAH 07	64	16	8	RL 07	32	16	≤1
TAH 09	250	8	Nt ^b^	RL 13	64	16	≤1
TAH 10	32	8	8	RL 20	16	16	4
TAH 11	64	16	8	RL 27	32	16	≤1
TAH 12	64	16	Nt ^b^	RL 32	32	16	≤1
TAH 13	64	16	4	RL 33	32	16	2
TAH 14	32	16	4	RL 36	32	8	2
TAH 15	32	16	4	RL 38	32	8	4

^a^ Fluconazole. ^b^ not tested. TAH: clinical isolate of *T. asahii*. RL clinical isolate of *C. parapsilosis.* The number beside the acronym represents the identification in the culture collections of the Laboratory of Applied Mycology Research of the Federal University of Rio Grande do Sul, Porto Alegre, Brazil.

An indication of the mechanism of action of the molecules could be observed when we assessed the compounds’ activity against *T. asahii* TAH06 and *C. parapsilosis* RL01 isolates. Both isolates are considered resistant to fluconazole according to the parameters established by the Clinical and Laboratory Standards Institute (CLSI) [18], that determines that isolates presenting a MIC for fluconazole greater than 8 μg/mL are considered resistant. Compound **7b** showed a MIC of 16 μg/mL for both isolates, so this activity may indicate differences in relation to the mechanism of action of fluconazole.

From the results of the MIC determination and the observation of distinct and diverse activities of fluconazole our efforts were next focused on the investigation of the mechanism of action of the active compounds.

### 2.3. Mechanism of Action

For the evaluated compounds, three tests were performed to investigate their mechanisms of action as described below.

#### 2.3.1. Evaluation of the Action on Cell Wall Stability: Sorbitol Protection Assay

The fungal cell wall serves as a protective barrier, preventing osmotic bursting of the cells. The cell wall is, therefore, essential for growth and fungal viability in a hypotonic environment. This is unnecessary if the fungi are protected with an osmotic support under specific conditions, so even if the cells have their cell walls damaged by molecules that inhibit its synthesis, they will continue to grow. Sorbitol was used in this experiment as osmotic protector [19]. The new MIC of these molecules was determined with a YNB environment supplemented with glucose in parallel experiments with and without the addition of 0.8 M sorbitol. The fungi treated with anidulafungin, an antifungal that acts on the cell wall synthesis, started to grow after incubation for 7 days. As shown in Table 4, the derivatives **13a** and **7b** did not have activity on the cell wall of the isolates, because the MIC remained the same in the reading at 2 and 7 days and in the environments with and without sorbitol.

**Table 4:** MIC in µg/mL of the compounds **13a** and **7b**, in the presence and absence of 0.8 M of sorbitol in *C. parapsilosis* RL 33 and *T. asahii* TAH 10.

Compounds	2 Days		7 Days	
	−/Sorbitol	+/Sorbitol	−/Sorbitol	+/Sorbitol
Anidulafungin	<1.0	<1.0	<1.0	>125
**7b**	16	16	16	16
**13a**	32	32	32	32

#### 2.3.2. Evaluation of the Action on Cell Membrane Stability: Cellular Leakage Assay

**Figure 1:**
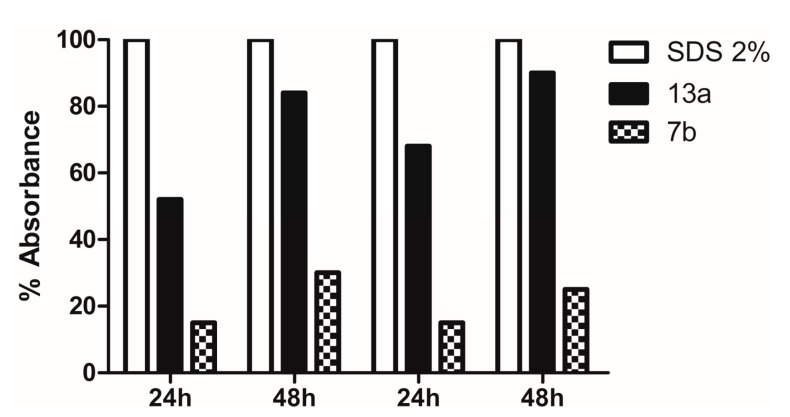
Release of intracellular components (absorbance read in 260 nm) of *C. parapsilosis* RL 33 and *T. asahii* TAH 10 treated with the compounds **13a** and **7b** and sodium dodecyl sulfate (SDS).

When there is damage to the fungal cell membrane, cellular components such as nucleotides spill out of the cell. These compounds exhibit a strong absorbance at 260 nm thus allowing its quantification, and through these data it is possible to assess the extension of the damage to the fungal membrane, as shown in Figure 1.

Taking the absorbance reading of 2% sodium dodecyl sulfate (SDS) as standard of 100% of cell lysis we compared the results of the tested compounds. The compound **13a** showed an absorbance of 90% of the standard after 48 h, leading to the conclusion that it causes strong damage to the cell membrane stability for both isolates. However, the compound **7b** appears to cause damage to the membrane, but in a more moderate manner when compared with the standard and with the former.

#### 2.3.3. Evaluation of Action on Membrane Ergosterol: Ergosterol Effect Assay

**Figure 2:**
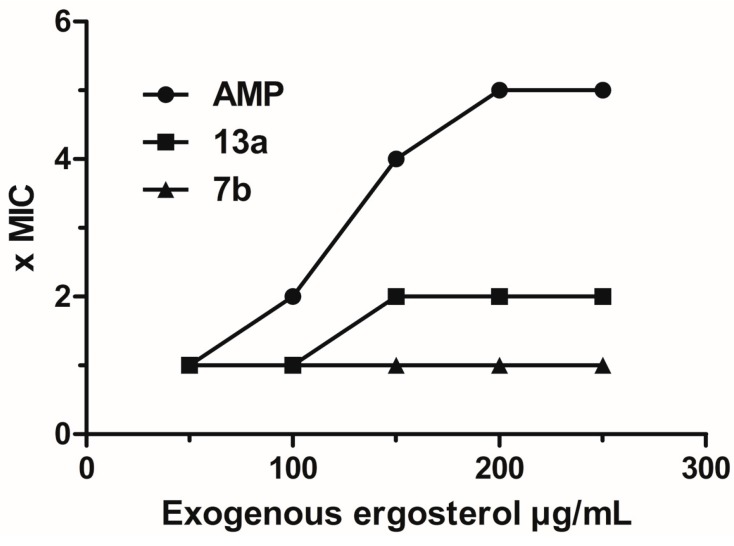
Effect of exogenous ergosterol (50–250 μg/mL) in the MIC of compounds **13a**, **7b** and amphotericin B (AMP) against *C. parasilosis* RL33 and *T. asahii* TAH10.

To determine if the damage to cell membrane occurs by binding to sterols of the membrane, the MIC of these compounds was determined again with and without the addition of ergosterol. In this test we could evaluate if the activity of the derivative is due to binding to membrane ergosterol, once exogenous ergosterol is added this prevents the binding of the compounds to endogenous ergosterol. As consequence, in positive cases the MIC increases in the presence of exogenous ergosterol. The standard for this test was amphotericin B, a drug that acts through this mechanism. As noted in Figure 2, none of the derivatives showed a significant increase of the MIC, which led us to believe that they have a mechanism of action that interferes with the cell membrane, but that does not involve an interaction with ergosterol.

### 2.4. Cell Viability and Genotoxicity

**Figure 3:**
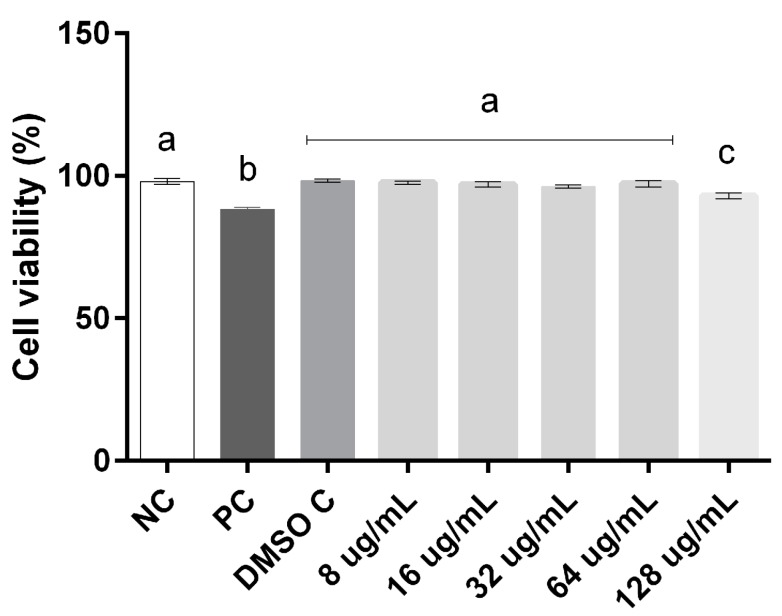
Percentage of cells in cell viability assay in human leukocytes exposed to different concentrations of **13a**. Data were analyzed by one-way ANOVA followed by Tukey test for multiple comparisons and expressed average ± SEM, with *p* < 0.001; ^a, b, c^ the superscript letters indicate statistical difference between groups; NC = negative control, DMSO C = DMSO control; PC = positive control.

The cytotoxicity and genotoxicity were evaluated in order to elucidate the concentrations capable of inducing cellular damage to both compounds. Our results demonstrate that compound **7b** showed toxicity at concentrations that had activity against yeasts and **13a** exposure ranging from 8 to 128 µg/mL had no effect on human leukocyte cell viability when compared to the negative control (*p* < 0.001) (Figure 3).

**Figure 4:**
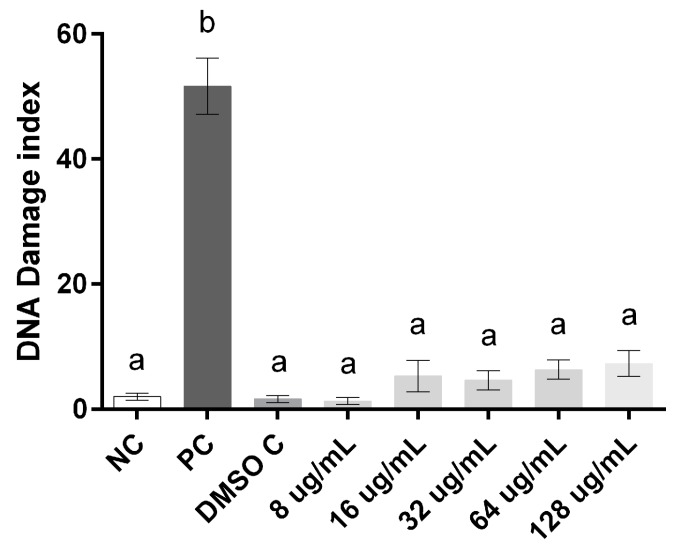
Index of DNA damage to human leukocytes exposed to different compound concentrations of **13a**. Data were analyzed by one-way ANOVA followed by Tukey test for multiple comparisons and expressed average ± SEM, with *p* < 0.001. ^a, b^ the superscript letters indicate statistical difference between groups; NC = negative control, DMSO C = DMSO control; PC = positive control.

Regarding the evaluation of damage to leukocyte DNA after exposure to compound **13a**, the comet assay demonstrated that the exposure concentrations tested were similar to the level of damage index found in both negative and DMSO controls, which leads us to suggest that this compound is not able to induce oxidative DNA damage in human leukocytes at the range of concentrations evaluated (Figure 4).

## 3. Experimental Section

### 3.1. General Information

All reactions requiring an inert atmosphere were carried out in a pre-dried apparatus under a nitrogen atmosphere. The reactions were monitored by thin layer chromatography (TLC), performed on silica gel 60 F254 plates (Merck, Kenilworth, NJ, USA). Visualization was achieved using a UV lamp at a wavelength of 254 nm. For the analysis of absorbance at 260 nm, a DU-600 spectrometer (Beckman, Brea, CA, USA) was used.

^1^H- and ^13^C-NMR spectra were recorded with an Inova 400 and a VNMRS 400 spectrometer (Varian, Palo Alto, CA, USA). Chemical shifts are shown in parts per million with tetramethylsilane (TMS) as a reference. HR-EI-MS spectra were recorded with an UltrOTOF mass spectrometer (Bruker Daltonics, São Paulo, Brazil).

The aldehydes **1a–7a** and other reagents were purchased from a commercial supplier (Sigma-Aldrich Co.). Compounds, **6b** and **14b** are unpublished and the corresponding structural elucidation data are presented below. The other products are known compounds and were identified by comparison of their physical and spectral data with those of authentic samples.

### 3.2. Chemistry

#### 3.2.1. General Procedure for the Preparation of Derivatives **8a–12a**

The *O*-methylation was performed by reaction of aldehydes **1a–7a** with 4 equiv. of CH_3_I and 3 equiv. of K_2_CO_3_ to protect each hydroxyl group in acetone with heating at 80 °C for 12 h in Schlenk flask [20]. Compounds were purified by column chromatography (cyclohexane/EtOAc, 9:1). Yields were **8a**, **9a** = 90%; **10a**, **11a** = 92% and **12a** = 89%.

#### 3.2.2. General Procedure for the Preparation of Derivatives **13a–14a**

The brominated derivative (2-bromopyridine for **13a** and 1-bromo-4-nitrobenzene for **14a**) 1.1 equiv. of 4-formylphenyl boronic acid, Pd(OAc)_2_ 2 mol % and PPh_3_ 4 mol % were added to a Schlenk flask. Then, under an inert atmosphere methanol/THF 1:1 (2mL/mmol) and 2 equiv. of KOH were added. The system was stirred for 24 h at 60 °C [21]. Compounds were purified by column chromatography (cyclohexane/EtOAc, 9:1) in yields of 80% (**13a**) and 75% (**14a**).

#### 3.2.3. General Procedure for the Preparation of Derivatives **1b–14b**

The aldehydes **1a–14a** were added to 1 equiv. of *tert*-butylcarbazate in a Schlenk flask under an inert atmosphere in a 1:1 toluene/isopropanol mixture as solvent. The system was stirred for 2 h at 85 °C and then stirring was continued for more 14 h at r.t. The precipitates were collected by filtration, recrystallized and dried, yielding the products in 90%–95% yields [22].

*Tert-Butyl(2Z)-2(3,5-dihydroxybenzylidene)hydrazinecarboxylate* (**6b**). White powder; 91% yield; ^1^H-NMR (CD_3_OD) δ 7.7 (s, 1H), 6.6 (d, *J* = 2.2 Hz, 2H), 6.3 (t, *J* = 2.2 Hz, 1H), 1.5 (s, 9H); ^13^C-NMR (CD_3_OD) δ 158.4, 154.1, 144.3, 136.3, 105.1, 103.8, 80.6, 80.5, 27.3. HRMS (ESI-MS, *m*/*z*), (C_12_H_16_N_2_O_4_) calcd [M+H]^+^ 253.2664; found 253.2677.

*Tert-Butyl(2Z)-2-[(4′nitrobiphenyl-4-l)methylene]hydrazinecarboxylate* (**14b**). White powder; 90% yield; ^1^H-NMR (CD_3_OD) δ 8.6 (s, 1H), 8.62 (d, *J* = 2.2 Hz, 2H), 7.8 (t, *J* = 2.2 Hz, 1H), 7.5 (s, 2H), 7.2 (s, 2H) 1.6 (s, 9H). ^13^C-NMR (CD_3_OD) δ 155.5, 148.1, 147.1, 146.9, 145.0, 128.8, 127.4, 123.9, 81.0, 28.4. (ESI-MS, *m*/*z*), (C_18_H_19_N_3_O_4_) calcd [M + H]^+^ 342.3613; found 342.3650.

#### 3.2.4. General Procedure for the Preparation of Derivatives **1c**, **8c–14c**

The hydrazones **1b**, **8b–14b** were subjected to catalytic reduction with Pd/C 10% (0.2 equiv.) and HCOONa (1.8 equiv.) dissolved in a 5:1 mixture of ethanol/water (1 mL/mmol). The mixture was stirred for 1.5 h at 60 °C and then cooled down to a temperature below 40 °C and stirred for 12 h [22]. At the end of the reaction, the solution was extracted with EtOAc, dried over MgSO4, and concentrated *in vacuo*. The residue was purified by column chromatography (cyclohexane/EtOAc, 8:2) in yields of 70% (**1c**); 70% (**8c**, **9c**, **12c**) and 65% (**11c**, **13c**).

### 3.3. Antifungal Activity

#### 3.3.1. Isolates

The set of clinical isolates of *Candida albicans*, *Candida krusei*, *Candida glabrata*, *Candida tropicalis* and *Candida lusitaneae*, *Candida parapsilosis* and *Trichosporon asahii* was obtained from the culture collections of the Laboratory of Applied Mycology Research of the Federal University of Rio Grande do Sul, Porto Alegre, Brazil.

#### 3.3.2. Antifungal Activity Screening Assay

Antifungal activity screening assay was carried out using the broth microdilution method. In a 96-well plate samples were added to a final concentration of 500 µg/mL incubated at 35 °C for 48 h. The molecules were considered active when there was no apparent fungal growth [23].

#### 3.3.3. Minimal Inhibitory Concentration

Minimal inhibitory concentration (MIC) of the compounds was determined by the broth microdilution method according to the M27-A3 documents published by the Clinical Laboratory and Standards Institute [18] with RPMI-MOPS (RPMI 1640 medium containing L-glutamine without sodium bicarbonate (Sigma-Aldrich Co.) buffered to pH 7.0 with 0.165 mol/L MOPS buffer (Sigma). The concentrations of the extracts ranged from 0.5 to 256 µg/mL. The MIC was defined as the lowest concentration of compounds at which the microorganism tested did not demonstrate visible growth.

### 3.4. Mechanisms of Action

#### 3.4.1. Sorbitol Protection Assay

The MIC values were determined using the broth microdilution procedure according to the CLSI [18]. The cells were inoculated at a final concentration of 2 × 10^3^ CFU/mL and cultured in Yeast Nitrogen Base (Difco, Detroit, MI, USA) with 0.5% glucose and incubated at 30 °C. Duplicate plates containing the test samples were prepared and in one of them 0.8 M sorbitol was added as an osmotic support environment. The plates were read at 2 and 7 days [19,24,25]. Anidulafungin (Ecalta^®^) was purchased from Pfizer^®^ (New York City, NY, USA; each 1.0 g of injectable product contains 100 mg of anidulafungin) and used as a control for this mechanism assay.

#### 3.4.2. Cellular Leakage Assay

Cells were cultivated in MOPS tampon (Sigma-Aldrich Co., St. Louis, MO, USA), pH 6 to 10^7^ CFU/mL and transferred to tubes. The compounds were added at a final concentration = MIC. Sodium dodecyl sulfate, (SDS, 2%) was used as reference compound that produces 100% of cell loss. During incubation at 30 °C, aliquots were taken at timed intervals (24, 48 h) [19,25].

#### 3.4.3. Ergosterol Effect Assay

Performed by CLSI standard broth microdilution in duplicate, in the absence and presence of different concentrations (50–250 µg/mL) of ergosterol (Sigma-Aldrich Co.) added to the environment. Amphotericin B (AMP, 972 μg/mg kindly supplied by Cristália^®^, Itapira, Brazil) was used as a control for this mechanism assay. The MIC was determined after 24 h of incubation [19,25].

### 3.5. Cytotoxicity

For the assays cell viability and alkaline comet samples were used in triplicate. The division of the groups for the assays was designed as follows: Negative control, which consisted of adjusted leukocyte suspension (LS, 130 cells/mm^3^) in PBS tampon pH 7.4; Positive control, consisting of the LS and the addition of 4 mm H_2_O_2_; DMSO control, consisting of the LS and addition of DMSO 0.5%. In both experiments the compounds were tested at 5 concentrations (8, 16, 32, 64, 128 µg/mL) starting from the active concentrations in determining the MIC. All groups were incubated for an hour and subjected to inversion every 10 min at an environment temperature of 25 °C.

#### 3.5.1. Cell Viability

To evaluate the cytotoxicity the assay of cell viability was performed, considering the loss of membrane integrity by the method of trypan blue [26]. Briefly, 100 µL of LS after incubation was mixed with 100 µL of trypan blue 0.4% for 8 min. Cell viability was determined microscopically (magnification 400×) in a Neubauer chamber categorizing two scores: viable cells, which appear discolored or slightly colored by blue; and dead cells which are colored by blue. For this test, 300 cells were counted for each analyzed sample.

#### 3.5.2. Genotoxicity

To evaluate the genotoxicity it was used the alkaline comet assay [27], and in accordance to the guidelines for performing the comet assay [28]. Blades analysis was performed on microscope counting 100 cells per blade and classified in damage levels from 0 (no damage) to 4 (maximum damage). The mean obtained from each treatment allowed the calculation of the damage rate, where the number of cells was multiplied by the level of damage related, according to the scale of damage [28].

### 3.6. Statistical Analysis

All data were treated by analysis of variance (ANOVA) and complemented by Bartlett Test, when relevant, accepting *p* < 0.05.

## 4. Conclusions

In this study thirty-five compounds, among them 28 synthesized in yields above 80%, were evaluated for *in vitro* antifungal activity against seven species of yeasts. Two molecules, **13a** and **7b**, stood out, with MIC values between 8 and 32 μg/mL, against clinical isolates of *C. parapsilosis* and *T. asahii*, species of great epidemiological importance and of great severity in infections. It was further observed that such compounds demonstrated great effect on cell membrane stability, but no interaction with ergosterol. Compound **13a** did not show cytotoxicity against leukocytes or genotoxic effects. It has been shown, therefore, that this molecule presents promising activity against emerging yeasts, and could be of great importance in the development of alternatives for the treatment of such infections.
